# Steam Sterilization of Equine Bone Block: Morphological and Collagen Analysis

**DOI:** 10.1155/2018/9853765

**Published:** 2018-08-13

**Authors:** R. Lo Giudice, G. Rizzo, A. Centofanti, A. Favaloro, D. Rizzo, G. Cervino, R. Squeri, B. G. Costa, V. La Fauci, G. Lo Giudice

**Affiliations:** ^1^Department of Clinical and Experimental Medicine, Messina University, Italy; ^2^Department of Biomedical and Dental Sciences and Morphofunctional Imaging, Messina University, Italy

## Abstract

**Introduction:**

The use of equine bone blocks is widely reported for bone augmentation techniques. The block must be shaped according to the form of the defect that should be regenerated. The shaping could be performed by hand before or during the surgery, in a sterile ambient, or using a CNC milling machine that could not be sterile. The aim of our study was to evaluate if a steam sterilization could provide a medical grade sterilization of the blocks and to evaluate if bone microstructure and collagen structures change after different steam sterilization protocols provided by mainstream autoclave.

**Materials and Method:**

Two blocks of equine bone were divided into 16 samples. 1 sample was used as control and not submitted to any treatment. 15 samples were infected with a Streptococcus faecalis bacterial culture. The samples were singularly packed, randomly divided into 3 groups, and submitted to autoclave sterilization on the same device. The groups were submitted to a sterilization cycle (Gr. A: 121°C, 1,16 bar for 20′; Gr. B:134°C, 2,16 bar for 4′; Gr. C: 134°C, 2,16 bar for 3.30 min.). 2 samples for each group were evaluated for the sterility. 3 samples for each group were observed at SEM to notice the macro- and microstructure modification and to confocal microscope to observe the collagen.

**Results:**

All samples were sterile. The SEM evaluation showed, in all groups, a preserved morphological structure. Confocal microscope evaluation shows that the collagen structure appears to be more uniform and preserved in group C.

**Conclusion:**

Data show that autoclave steam sterilization could be reliable to obtain sterilization of equine bone blocks.

## 1. Introduction

Bone regeneration is a reliable technique when the bone volume is not sufficient to provide a long-term stability of implant-supported prosthetic restorations and functional and aesthetical outcome. It is also indicated in the posttraumatic and oncologic reconstructive protocols [1].

The autologous bone is still considered the gold standard, harvested from extra or intraoral site [2–4].

The homologous fresh frozen bone has also been reported as effective thanks to its osteoconductive and it is potentially osteoinductive properties linked to its matrix contains growth factors such as bone morphogenetic protein (BMP) and vascular endothelial growth factor (VEGF) [5, 6].

However, in bone regeneration procedures, due to the increased morbidity, limited quantities available and the necessity of a second surgical site are widely used xenogenic materials of bovine, porcine, or equine origins [7, 8].

Xenografts, thanks to their chemical-physical characteristics similar to those of the human bone, show osteoconductive properties [9, 10].

Enzyme-deantigenic equine bone has been used successfully in several fields of oral surgery and implantology [11] including periapical cyst-removal management, periodontal defect correction [12], horizontal and vertical atrophic ridge reconstruction [13–17], and sinus augmentation [18–22].

Equine-derived biomaterials may be preferred to those of bovine or porcine origins for issues related to Transmissible Spongiform Encephalopathies. It is well known in fact that rabbits, dogs, and horses are the only mammalian species reported to be resistant to infection from prion diseases isolated from other species [23, 24].

In equine bone, an enzymatic process to preserve the native conformation of type I collagen equine bone is used while making the bone nonantigenic. The bone collagen matrix contains a high amount of growth factors such as IGF-II, TGF-beta, IGF-I, PDGF, bFGF, BMPs, and others [25] and this implies that the demineralized bone matrix (DBM) is able to stimulate the production of new bone tissue [25] when grafted in another species [26–28].

Regarding cellular behavior, it has been found that when osteoclasts were cultured on enzyme-deantigenic equine bone, they adhered on this material in greater numbers and exerted a more intense degrading activity [29] compared to inorganic bovine bone [30], possibly because of the presence of preserved collagen in the equine xenograft.

In vitro studies showed the effects of preserved bone's collagen on biological mechanisms related to bone regeneration. A randomized clinical trial found that enzyme-deantigenic equine bone led to a significantly greater amount of newly formed bone and a lower residual biomaterial, compared with inorganic bovine bone [31].

The bone substitutes may be used in different forms such as block or particulate along with the application of a long-lasting membrane to prevent soft tissue cells from invading the regenerating site (GBR) [32].

It has been reported that a bone block is a reliable material for regenerative procedures with predictable long-term positive results [33]. The space maintaining characteristics of the blocks can overcome the poor three-dimensional stability of the particulate substitutes offering an adequate mechanical support to the overlying tissue [16, 34, 35].

Allograft and xenograft bone substitutes underwent to procedures that greatly lower the potential risk of transmission of bacteria, viruses, and prions.

Different sterilization techniques are used for autologous tissues including gamma irradiation [36], ethylene oxide gas [37], thermal treatment with moist heat [38], beta-propiolactone [39], chemical processing [40], and antibiotic soaks [41].

In xenografts, the microbial safety is generally achieved through gamma or electron-beam (e-beam) irradiation [42]. E-beam irradiation may be preferred to gamma because the more precise dose control allows a shorter irradiation time [43, 44].

In the common dental practice, the autoclave is used to obtain a high-grade sterility of various kind of material [45–47].

The aim of our research was to evaluate if different autoclave sterilization protocols may modify the macroscopical and microscopical bone structure of an equine block and if this protocols will affect the collagen matrix.

## 2. Materials and Method

Two equine bone blocks (10x20x5 mm) with collagen (OX Block, Osteoxenon, Bioteck, Italy) were divided into 16 samples of cubic shape (5x5x5 mm) using a stainless steel bone cutter Lindemann bur (Komet, Komet It Srl, Italy).

### 2.1. Sterilization Test

One sample was used as control and not submitted to any treatment.

15 samples were infected with a bacterial culture.

The Streptococcus faecalis, used for sterilization test, was previously isolated from a pharyngeal buffer and incubated, under standardized conditions, for 24 h. in sterile thioglycollate broth at 37±2 C.

The pH-values of the broths were measured at the beginning and end of each incubation cycle.

1 ml of thioglycollate broth contained 1 × 10^5^ CFU/ml Streptococcus faecalis colonies.

Each sample was contaminated by using sterile micropipettes, with 0,5 ml of thioglycollate broth.

The 15 samples were randomly divided in 3 groups and submitted to autoclave sterilization in the same device (Euronda E9 Med, Euronda, Italy).

A group was submitted to a sterilization cycle at 121°C, 1,16 bar for 20 min. and 15 min. of drying.

B group was submitted to a sterilization cycle at 134°C at 2,16 bar for 4 min. of sterilization and 15 min. of drying.

C group was submitted to a sterilization cycle at 134°C at 2,16 bar for 3.30 min. of sterilization and 5 min. of drying.

Two samples for each group were evaluated for the sterility.

1 sample per group was incubated under standardized conditions for 24 h in thioglycollate broth at 37±2°C.

1 sample per group was incubated under standardized conditions for 24 h in normal saline solution at 37±2°C.

To evaluate the sterilization effects, 18 *μ*l of thioglycollate and 18 *μ*l of normal saline solution were collected and incubated for 24 h at 37±2°C in CLED agar and ESCULINA agar, for the bacterial count.

After 24 h of incubation the bacterial presence was evaluated by a conventional biochemical test and colonies were counted and interpreted as colony-forming units.

### 2.2. Structural Evaluation

After autoclave steam sterilization, the 9 samples that were not evaluated for sterility underwent to SEM (Phenom Pro 5, Phenom-World B.V., Eindhoven, Netherlands) observation to directly observe the macroscopical and microscopical structure.

Each sample was fractured to observe an uncut surface.

All 9 samples underwent to optical observation to evaluate the macroscopical structural modification.

Three randomly areas of each surface were observed at the same magnification and the microstructural changes were recorded.

After the SEM observation the 12 samples underwent to the confocal microscope observation to evaluate the collagen presence. The uncut and unfractured surface was observed.

### 2.3. Scanning Electron Microscopy Protocol

After fixation in 2% glutaraldehyde, specimens were dehydrated in ethanol and amyl acetate and then were dried at critical point in a Balzers critical point drier using liquid CO2. The bone fractured surfaces were mounted on stub and platinum coated with a sputtering system “Plasma Sciences CrC-100 Turbo Pumped” and observed by Phenom G2 pro scanning electron microscope.

### 2.4. Confocal Microscope Protocol

After fixation in 2% glutaraldehyde and rinsing in phosphate buffer 0,13 mol/L, pH 7.3, specimens were decalcified in 4.13% Ethylenediaminetetraacetic acid, pH 7.2, dehydrated in ethanol, and embedded in paraffin. A Leica microtome (Leica RM2255, Leica, Leica Biosystems, MI, Italy) was used to obtain eight-micrometer-thick sections.

The sections were treated with the following antibodies: mouse monoclonal anticollagen I (diluted 1:1000; Sigma-Aldrich) which were demonstrated with IgG-Texas Red conjugated anti-rabbit (1:100 dilution; Jackson ImmunoResearch Laboratories, West Grove, PA, USA) [48, 49].

Negative controls were carried out by treating the sections only with the secondary antibody. The samples were observed with the Zeiss LSM 510 confocal microscope (Zeiss LSM 510, Carl Zeiss S.p.A., MI, Italy). The “display profile” function of the laser scanning microscope was used to show the intensity profile across an image along a freely selectable line [50].

## 3. Results

### 3.1. Sterilization Test

The sterility was achieved in all samples, considering a SAL (Sterility Assurance Level) of 10^−6^.

### 3.2. Scanning Electron Microscopy Evaluation

**Figure 1:**
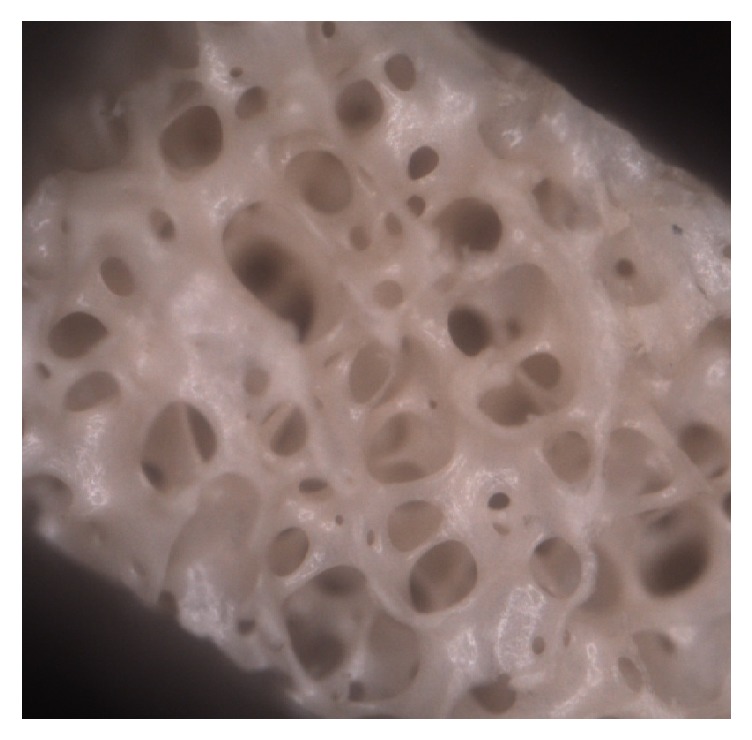
Group A: spherical and ovoid cavities delimitated by trabeculation (42x).

Group A: image shows a defined bone architecture with cavities of spherical shape delimited by a dense trabeculation (42x) (Figure 1).

**Figure 2:**
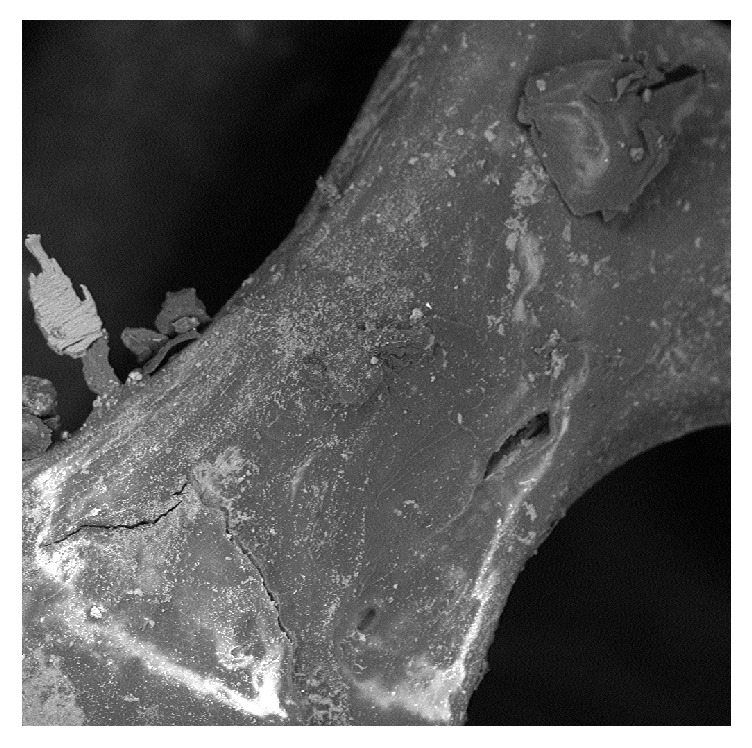
Group A: an intact trabecula with some residues on it.

It is possible to observe in detail a trabecula, which delimits two cavities, and it is possible to see the presence of some residues of different shapes and sizes. Bone morphology appears to be well preserved (1100x) (Figure 2).

**Figure 3:**
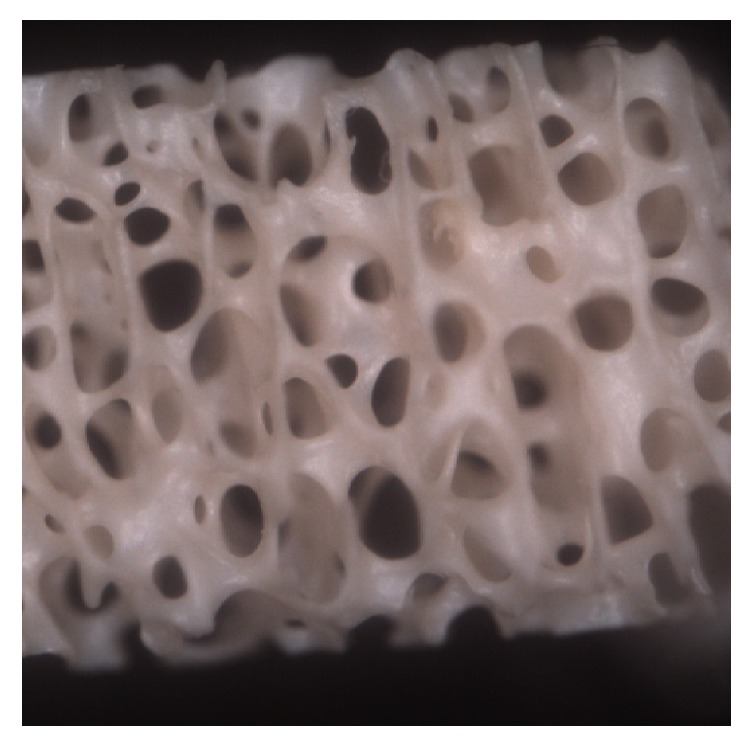
Group B: morphologically well-preserved communicant cavities of ovoid and spherical shape delimitated by a dense trabeculation (40x).

**Figure 4:**
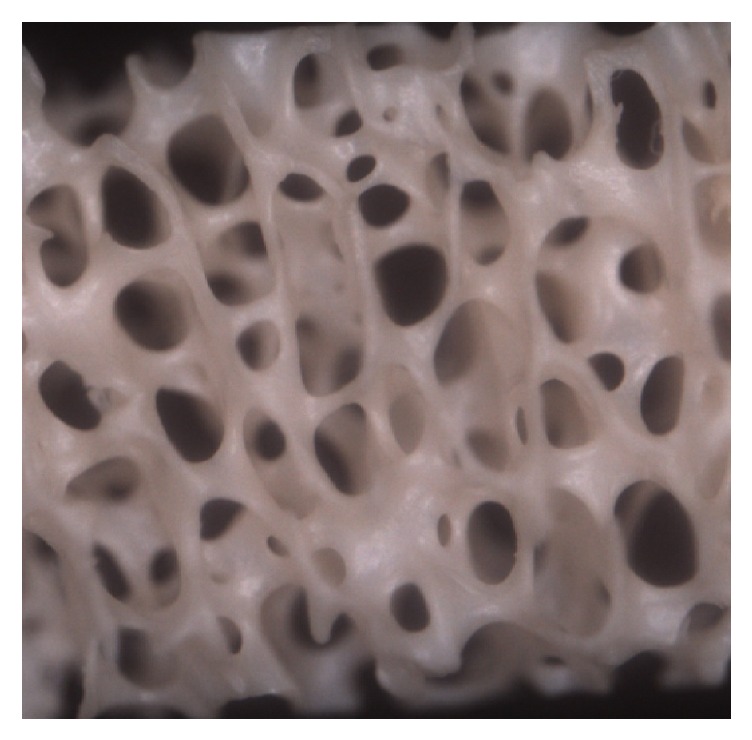
Group C: morphologically well-preserved communicant cavities of ovoid and spherical shape delimitated by a dense trabeculation (40x).

Group B and group C: images show a cavity of ovoid and spherical shape delimitated by a dense trabeculation (40x) (Figures 3 and 4).

**Figure 5:**
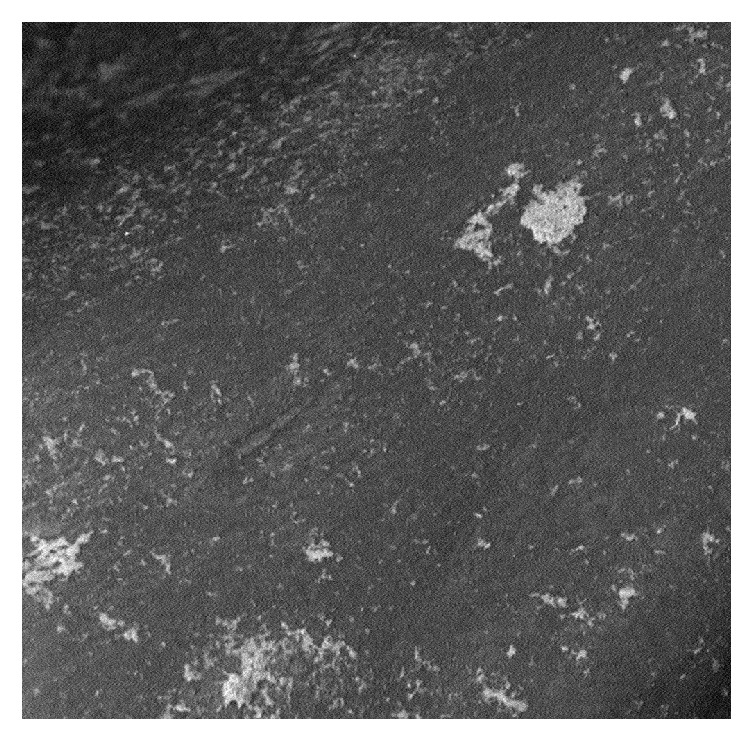
Group B: a trabecula with sand-like residuals on it (2500x).

**Figure 6:**
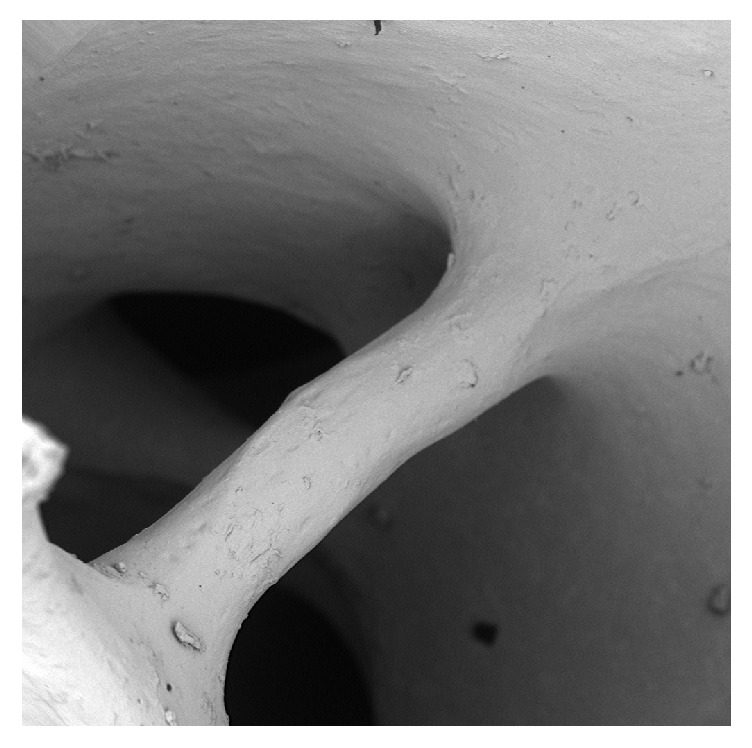
Group C: a well-preserved trabecula with smaller communicating cavities; the trabecula surface appears to be well preserved (420x).

Increasing the magnification, it is possible to observe a trabecula with smaller communicating cavities; the trabecula surface appears to be well preserved (2500x) (Figures 5 and 6).

### 3.3. Confocal Microscope Evaluation

**Figure 7:**
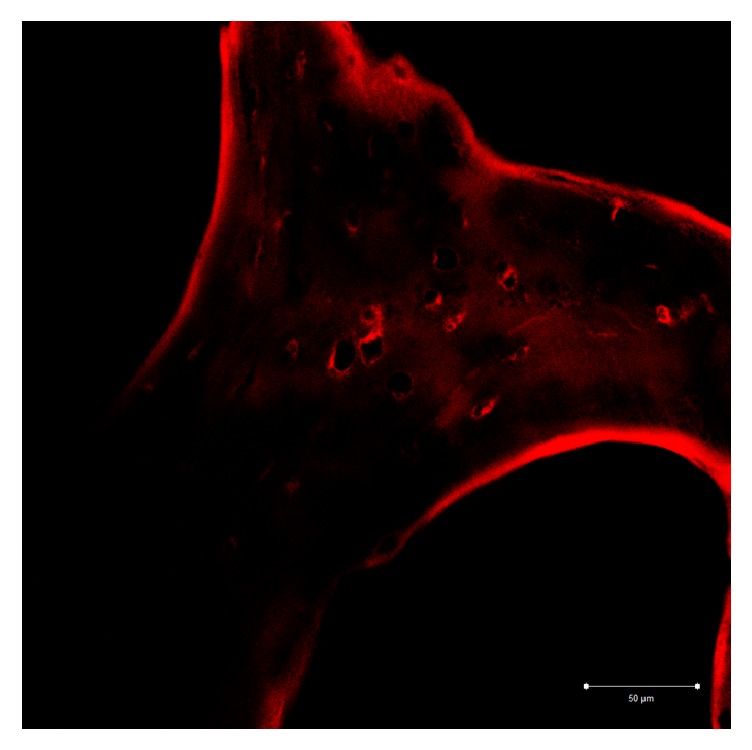
Group A: a continuous fluorescence pattern of type 1 collagen is noticeable on the peripheral surface of the trabecula. A light fluorescence is evident.

Group A: the image shows that type 1 collagen of the sample presents a fluorescence pattern that is located at the periphery of the trabecula continuously. A feeble fluorescence can be observed at the edges of some gap (Figure 7).

**Figure 8:**
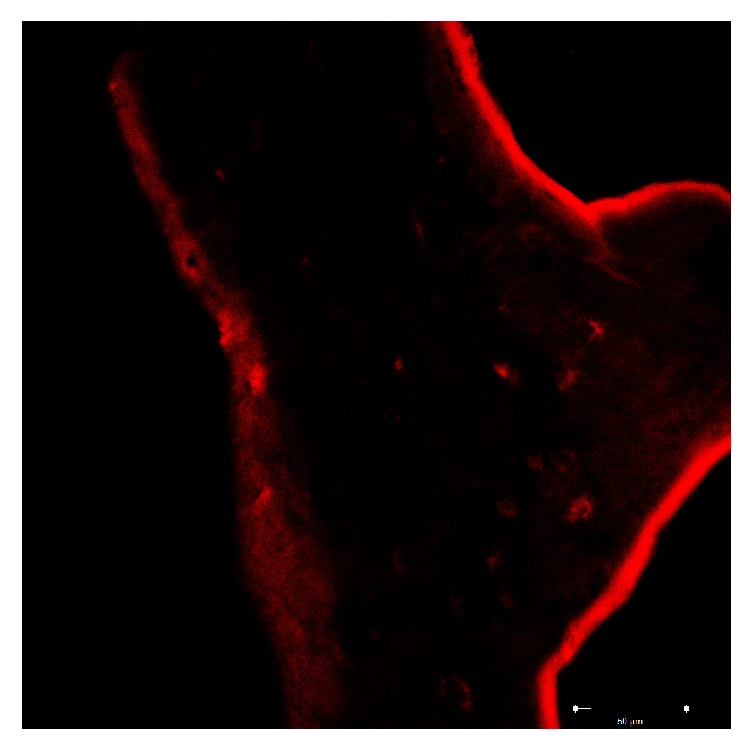
Group B: the fluorescence pattern of the type 1 collagen is located in the peripheral of the trabecula and appears variously intense.

Group B: the images show that type 1 collagen of the sample presents a fluorescence pattern that is located at the periphery of the trabecula sometimes continuous and intense, sometimes weak and widespread. A feeble fluorescence is observed at the edges. This group appears to be similar to group A (Figure 8).

**Figure 9:**
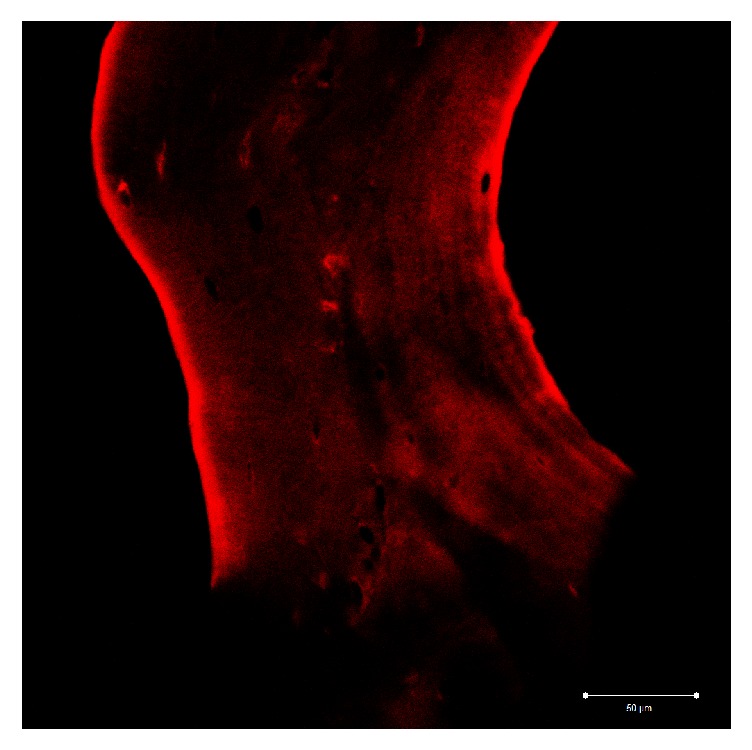
Group C: the fluorescence pattern of type 1 collagen is evident on trabecula and appears continuous and uniform. The whole trabecula shows a fluorescence pattern more marked near the lacuna.

Group C: the image shows that type 1 collagen of the sample presents a fluorescence pattern that is located at the periphery of the trabecula in a continuous and uniform manner. The entire trabecula has a weak diffuse fluorescence pattern with a circumferential course, sometimes more marked around the gaps (Figure 9).

Display profile shows the fluorescence intensity along a selected line.

**Figure 10:**
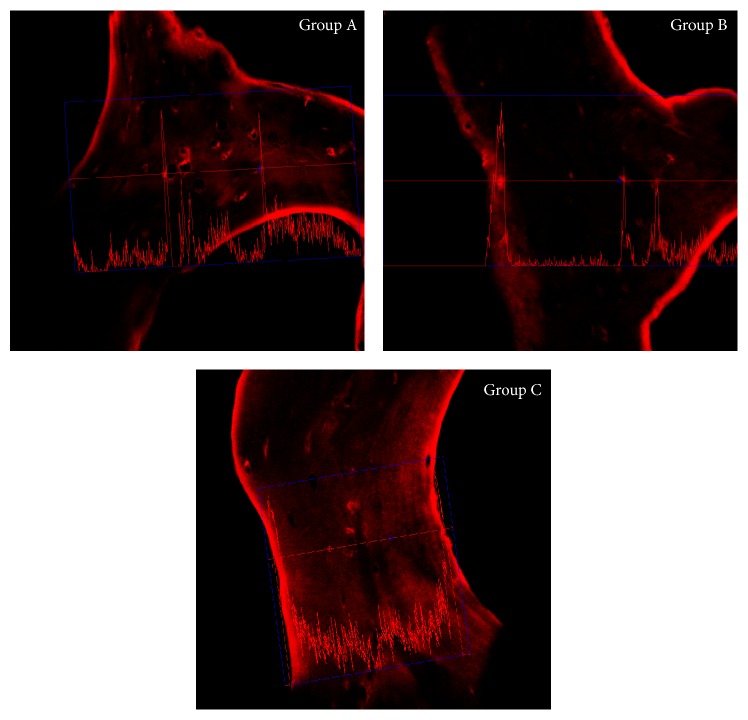
Display profile analysis.

The fluorescent signal intensity appears more pronounced and uniform in group C when compared to groups A and B. Comparing group A and group B a greater signal intensity is evident in group A (Figure 10).

## 4. Discussion

The bone augmentation procedure is widely studied in literature and commonly used in clinical practice and might be mandatory in different clinical situations in both medicine and dentistry [5].

Materials different for origin and composition were deeply studied by the scientific community and the differences between the particulate form and the bone block are commonly known.

Several studies analyzed the employment of bone equine-derived block containing collagen for ridge augmentation [51–53]. These researches revealed the advantageous capacity to maintain the dimensions of the augmented ridge. Benic et al. demonstrated that the GBR technique performed with equine block with collagen membranes (CM) resulted in the most favorable outcomes compared to bovine block and bovine granulate with CM [35].

It is mandatory to shape the bone block according to the defect that should be filled. The shaping could be performed by the industry that, according to a CBCT provided by the clinician, could send a preshaped and sterilized block or a handmade “in-house” shaping before or during the surgery. The handmade shaping has, however, a lack of precision related to the method of shaping. To overcome this issue a CNC milling machine could be used. This machine has a micrometer precision and in addition to a three-dimensional reconstruction could provide the clinician a precise model; however, due to its characteristic, the sterility requirement is not satisfying and must be achieved after shaping, using other techniques [54].

Cusinato et al. [55] demonstrated the efficacy of hydrogen peroxide treatment and e-beam irradiation on viral clearance in equine-derived xenografts (pericardium membrane, collagen membrane, and cortical and cancellous bone grafts) spiked with three human viruses (Coxsackie virus B1, influenza virus type A with subtype H1N1, and herpes simplex virus type 1).

Our work shows how it is possible to obtain a satisfactory sterility of the graft, using a traditional device as the autoclave.

The microbiological analysis shows how, in agreement with the literature, the sterility was achieved in all samples.

Our research was also aimed at understanding if this procedure might modify the bone morphology and the collagen.

The eye observation of the samples shows a fairly dehydrated and a slightly lighter white surface when compared to the control sample.

The SEM observations between the control sample and the samples of the three groups underline how, at different magnifications, the macrostructure does not show substantial modifications [56].

In all samples, the bone structure appears to be well preserved and the morphology appears to be very similar. It is evident how the high temperature did not determine evident and significant modifications of the bone sample architecture.

The collagen matrix that is a particular characteristic of the equine bone graft and is preserved due to the impossibility of this species to be infected with prions appears to be variously maintained.

The confocal microscope immunofluorescence analysis shows how group C (131 fast) has the best-preserved collagen structure. This observation is also confirmed by the display profile analysis, which evaluating the fluorescence intensity underlines how the fluorescent signal appears to be more uniform and intense when compared to the other groups. When comparing A and B groups, the first one shows a stronger and a uniform fluorescence.

The temperatures reached in steam autoclave sterilization appear to be respectful of the collagen fibril that remains always below the thermal transitions temperatures [57, 58].

The collagen resistance to heat and pressure damage, so, appears to be time related. When a steam sterilization is performed a 134°C and 2,16 bar for 3.30 min. and 5 min. of drying protocol is suggested.

From the analysis of our data, autoclave steam sterilization method could be a reliable way to obtain sterilization of bone graft.

Giving the possibility of using a nonsterile CNC milling machine will open a new scenario of precision in bone grafting, letting the surgeon to obtain a perfectly shaped and sterilized graft wherever before the surgery.

## 5. Conclusion

The autoclave sterilization appears to be a reliable way to obtain a medical grade sterilization of the equine bone blocks and it does not affect the macro- and the microstructure and the collagen fiber.

Further studies are necessary to evaluate if there are any chemical modification and to evaluate if there are changes in the in vivo osteointegration process of the bone that underwent the steam sterilization protocol.

## Data Availability

The data (photos) used to support the findings of this study are available from the corresponding author upon request.
